# Interpretations on nomogram for predicting overall and cancer-specific survival in patients with postoperative follicular thyroid cancer in thyroidology

**DOI:** 10.1016/j.bjorl.2026.101826

**Published:** 2026-05-18

**Authors:** Ilker Sengul, Demet Sengul

**Affiliations:** a Giresun University, Faculty of Medicine, Thyroidology Unit, TR28100 Giresun, Turkey; b Giresun University, Faculty of Medicine, Division of Endocrine Surgery, TR28100 Giresun, Turkey; c Giresun University, Faculty of Medicine, Department of General Surgery, TR28100 Giresun, Turkey; d Giresun University, Faculty of Medicine, Department of Pathology, TR28100 Giresun, Turkey

Dear Editor,

The recent article by Liu et al.,^1^ establishing a nomogram for postoperative Follicular Thyroid Cancer (FTC) survival, offers a commendable statistical analysis of a substantial cohort, published in volume 92, *Braz J Otorhinolaryngol*. However, the reliance on the Surveillance, Epidemiology, and End Results (SEER) database necessitates a critical evaluation of the model's biological depth and its applicability in the current era of precision oncology. A significant limitation may remain the absence of molecular determinants, such as TERT promoter mutations and BRAF mutations. The 2022 World Health Organization, WHO, classification has increasingly integrated genomic signatures to define aggressive thyroid malignancies. A prognostic tool derived exclusively from clinicopathological variables ‒ notwithstanding its reported C-index ‒ risks incompleteness if it does not account for the mutational landscape that fundamentally drives distant metastasis in FTC. As such, one might consider whether the model’s predictive accuracy would remain robust if challenged by molecularly stratified cohorts. Furthermore, the inherent granularity of data within registries like SEER often precludes a nuanced distinction between minimally and widely invasive FTC. Of note, while the authors utilized “glandular invasion” as a variable, the specific degree of vascular invasion remains the most potent predictor of mortality in FTC. The lack of this refined histological detail suggests that the nomogram may reflect broad historical trends rather than the subtle biological indicators required for individualized patient management. Additionally, the inclusion of “marital status” as a prognostic factor warrants caution. While statistical significance was achieved, such sociodemographic variables frequently serve as surrogates for healthcare access and psychosocial support. Incorporating non-biological factors into a survival model may lead to overfitting within specific socioeconomic contexts, potentially limiting the model’s external validity across diverse global healthcare systems. Eventually, Liu and colleagues^1^ provide a valuable perspective on FTC outcomes. Nevertheless, the integration of prospective data, including molecular stratification, is essential before such a tool can be adopted as a definitive bedside instrument. This issue merits further investigation. We thank the authors for their worthy work in *Braz J Otorhinolaryngol* on a nomogram for FTC.

## ORCID ID

Ilker Sengul: 0000-0001-5217-0755

Demet Sengul: 0000-0002-0416-0621

## Ethics approval

Not applicable.

## Funding

None declared.

## Data availability

The datasets used and/or analyzed during this study are available from the corresponding author upon reasonable request.

## CRediT authorship contribution statement

**Ilker Sengul:** Conceptualization, Formal analysis, Investigation, Methodology, Project administration, Validation, Visualization, Writing – original draft, Writing – review & editing. **Demet Sengul:** Conceptualization, Formal analysis, Investigation, Methodology, Project administration, Supervision, Validation, Visualization, Writing – original draft, Writing – review & editing.

## Conflicts of interest

The authors declare no conflicts of interest.

## References

[1] Liu X., Chen S., Wu C. (2026). Nomogram for predicting overall and cancer-specific survival in patients with postoperative follicular thyroid cancer. Braz J Otorhinolaryngol.

